# Hexokinase I N-terminal based peptide prevents the VDAC1-SOD1 G93A interaction and re-establishes ALS cell viability

**DOI:** 10.1038/srep34802

**Published:** 2016-10-10

**Authors:** Andrea Magrì, Ramona Belfiore, Simona Reina, Marianna Flora Tomasello, Maria Carmela Di Rosa, Francesca Guarino, Loredana Leggio, Vito De Pinto, Angela Messina

**Affiliations:** 1 Department of Biological, Geological and Environmental Sciences, Section of Biochemistry and Molecular Biology, University of Catania, Italy; 2 National Institute of Biostructures and Biosystems (INBB), Italy; 3 Department of Biomedical and Biotechnological Sciences, University of Catania, Italy; 4 CNR Institute of Biostructures and Bioimaging, Catania, Italy

## Abstract

Superoxide Dismutase 1 mutants associate with 20–25% of familial Amyotrophic Lateral Sclerosis (ALS) cases, producing toxic aggregates on mitochondria, notably in spinal cord. The Voltage Dependent Anion Channel isoform 1 (VDAC1) in the outer mitochondrial membrane is a docking site for SOD1 G93A mutant in ALS mice and the physiological receptor of Hexokinase I (HK1), which is poorly expressed in mouse spinal cord. Our results demonstrate that HK1 competes with SOD1 G93A for binding VDAC1, suggesting that in ALS spinal cord the available HK1-binding sites could be used by SOD1 mutants for docking mitochondria, producing thus organelle dysfunction. We tested this model by studying the action of a HK1-N-terminal based peptide (NHK1). This NHK1 peptide specifically interacts with VDAC1, inhibits the SOD1 G93A binding to mitochondria and restores the viability of ALS model NSC34 cells. Altogether, our results suggest that NHK1 peptide could be developed as a therapeutic tool in ALS, predicting an effective role also in other proteinopathies.

Amyotrophic Lateral Sclerosis (ALS) is a fatal neurodegenerative disease characterized by the progressive degeneration of both upper and lower motor neurons^1^ (MNs). Over 160 missense mutations in the Superoxide Dismutase 1 (SOD1) gene account for 20–25% of familial ALS cases^2^, causing MNs death by accumulation of mutant SOD1 (mutSOD1) insoluble toxic aggregates^3^. Interestingly, mutSOD1 aggregates associate with the mitochondrial cytoplasmic side, especially in spinal cord MNs, producing mitochondrial failure^4^,^5^. Despite it is well known that mitochondria play a central role in bioenergetics metabolism, oxidative stress, apoptosis and axonal transport, the intimate underlying mechanism linking mitochondrial dysfunction in MNs of ALS patients or mice to mutSOD1 still remains elusive. Moreover, it is not yet well understood why MNs are more susceptible to the disease in comparison to other tissues.

A previous report showed that, only in the ALS rat spinal cord, mutSOD1 bind directly to the Voltage Dependent Anion Channel isoform 1 (VDAC1), reducing its channel activity^6^. VDAC1 is considered the master regulator of the mitochondria thanks to its crucial action of gate for metabolic and energetic substrates of the organelle^7^,^8^. Moreover, VDAC1 is the physiological receptor of Hexokinases^9^ (HKs). HKs catalyze the glucose phosphorylation and, by binding to VDAC1, they gain a preferential access to newly synthesized ATP. Furthermore, mitochondrial-bound HKs protect the cell from apoptosis, since they diminish VDAC1 propensity to interact with pro-apoptotic protein Bax^10^,^11^. Interestingly, reduced levels of HK1 were detected in spinal cord, compared to the brain^6^ or to other tissues^12^. Therefore, high levels of mutSOD1 binding to VDAC1 correlate with low levels of HK1 in spinal cord. Based on these evidences, we have hypothesized that in ALS a reduction of HK1 concentration increases VDAC1 propensity to interact with mutSOD1, producing thus mitochondrial dysfunction and cell death.

In this work, we demonstrate the intrinsic ability of SOD1 G93A, but not SOD1 wild type (SOD1 WT), to interact with VDAC1 and to compete with HK1 for binding VDAC1. We also show that a synthetic peptide, corresponding to the HK1 N-terminal region (NHK1 peptide) is able to interact with VDAC1, in *in vitro* and *in cellulo*, modifying its channel conductance. In addition, the NHK1 peptide inhibits the VDAC1-SOD1 G93A interaction in mitochondria purified from NSC34, a mouse motor neuron-like hybrid cell line. Moreover, the expression of the NHK1 peptide in NSC34 cell stably expressing SOD1 G93A, a recognized ALS cell model, recovers the mitochondrial malfunctioning linked to mutSOD1, and largely contrasts the cell death.

Overall, our data suggest that VDAC1 and HK1 play a key role in the bioenergetics metabolism of the MNs and could be considered as a promising therapeutic target in ALS.

## Results

### SOD1 G93A, at variance with SOD1 WT, binds VDAC1 with high affinity

The interaction between VDAC1 and SOD1 G93A in ALS model rat was reported^6^ and later questioned^13^. To validate our experimental plan, recombinant human VDAC1, SOD1 WT and SOD1 G93A were expressed, purified and refolded (Fig. S1), and the affinity of SOD1 proteins for mitochondria or VDAC1 was checked by means of several approaches.

**Figure 1:**
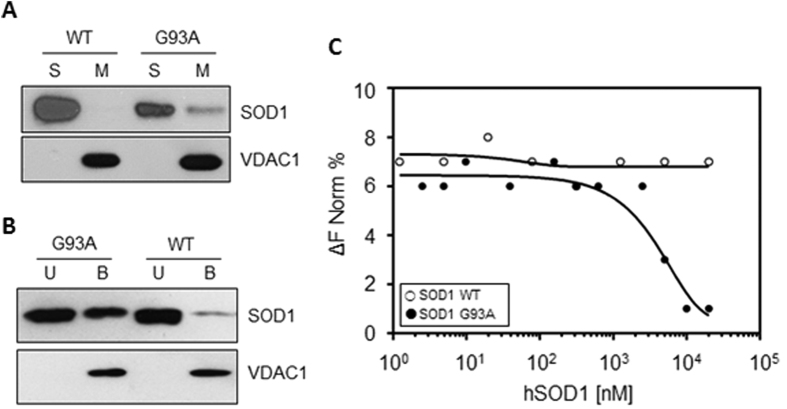
SOD1 G93A interacts with the cytosolic surface of mitochondria and VDAC1. (**A**) Representative western blot analysis (n = 3 independent experiments) of *in vitro* mitochondria-SOD1 proteins binding assay. Intact purified mitochondria were incubated with SOD1 WT or G93A and precipitated by centrifugation. VDAC1 was used as loading control. An aliquot of SOD1 G93A was found in mitochondrial pellet (M); on the contrary, SOD1 WT was exclusively present in the supernatant fraction (S). (**B**) Representative western blot analysis (n = 3 independent experiments) of *in vitro* binding assay between VDAC1 and SOD1 proteins. SOD1 G93A was found distributed between VDAC1-bound (B) or -unbound (U) fraction, while SOD1 WT was found almost exclusively in U fraction. (**C**) MST analysis of VDAC1-SOD1 interaction. Variation in normalized fluorescence (ΔFNorm%) was found exclusively for SOD1 G93A, indicating a specific interaction with VDAC1.

Intact isolated mitochondria from the motor neuron-like NSC34 cells were incubated with SOD1 WT or G93A, and mitochondrial membranes were precipitated by centrifugation. Then, the SOD1 concentration was revealed by western blot in mitochondrial membranes or in the supernatant fraction, using VDAC1 as a loading control. Results in Fig. 1A show that, while SOD1 WT was found exclusively in the supernatant, a fraction of SOD1 G93A co-precipitated in the mitochondrial pellet. According to the literature^3^,^6^, this data indicates that the recombinant SOD1 G93A, but not SOD1 WT, binds the mitochondria surface. The affinity of SOD1 proteins for VDAC1 was then studied by using an *in vitro* binding assay. Purified and refolded VDAC1 was immobilized on Ni-NTA magnetic beads and incubated with SOD1 proteins. Then, VDAC1-binding complexes were isolated by the application of a magnetic field. Figure 1B shows that SOD1 G93A was found distributed between VDAC1-bound and -unbound fraction, while SOD1 WT was almost exclusively in the unbound fraction. The VDAC1-SOD1 interaction was quantitatively assayed by Microscale Thermophoresis (MST) analysis. MST measures any variation in the thermal migration of a fluorescently labeled binding partner; changes of fluorescence in a heated spot of the protein solution is a function of increasing interacting protein concentration, and can be exploited to calculate the binding affinity coefficient (K_d_). The fluorescent-labeled VDAC1 was incubated with increasing concentrations of SOD1 proteins and the changes in fluorescence monitored. Again, as shown in Fig. 1C, while no fluorescence change was visible in the presence of SOD1 WT, the incubation with growing concentrations of SOD1 G93A produced fluorescence decrease, indicating that SOD1 G93A specifically interacts with VDAC1. Depletion curve was used to calculate the K_d_, which was estimated 4,81 μM.

Overall, the results showed here indicate that the mutant SOD1 G93A specifically interacts with the cytosolic surface of purified mitochondria and with the purified VDAC1 with high affinity. The SOD1 WT is instead unable to bind mitochondria and VDAC1, confirming the data in the literature^6^.

### SOD1 G93A modulates VDAC1 channel activity

A most relevant VDAC feature is the voltage dependence^14^. VDAC1 is characterized by a typical conductance of 4 nS in 1 M KCl, at low positive or negative voltages (±10 mV). In these conditions, the channel stays stably in an open and high-conducting state. However, raising the voltage, already at ±20–30 mV, VDAC1 switches rapidly to a closed and low-conducting state, where it can remain for quite a long time^14^,^15^,^16^. The ability of SOD1 proteins to interfere with VDAC1 activity was analyzed in terms of conductance perturbation.

**Figure 2:**
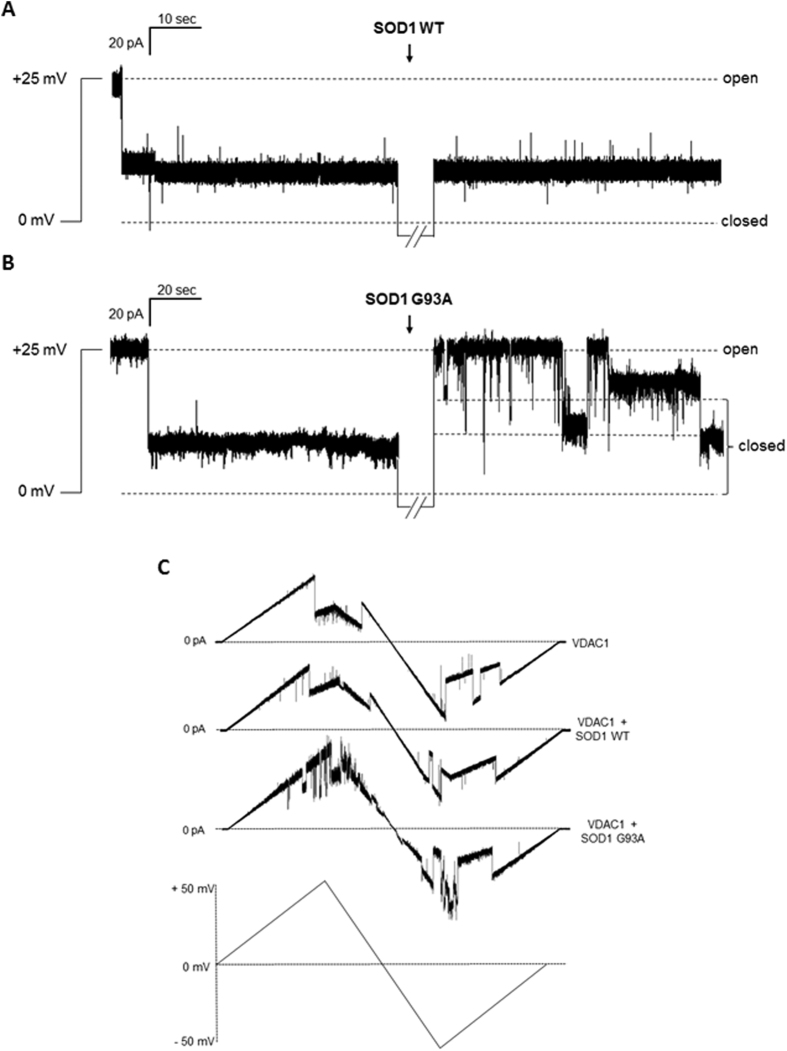
Electrophysiological characterization of VDAC1-SOD1 interaction at the PLB. (**A**) A representative trace of single channel analysis of VDAC1 conductance at +25 mV in 1 M KCl before and after the addition of SOD1 WT (n = 4). VDAC1 stably switched to the closed state depending on the applied voltage. Presence of SOD1 WT did not affect the channel conductance, since VDAC1 remained stable in its typical closed state. (**B**) A representative trace of single channel analysis of VDAC1 recorded using same condition in (**A**) before and after the addition of SOD1 G93A (n = 4). Presence of SOD1 G93A promoted instability of VDAC1 conductance. (**C**) Triangular curve of different experiments performed in a symmetric 1 M KCl solution with VDAC1 inserted in the membrane (n = 15) (upper curve) or upon addition of SOD1 WT (n = 5) (middle curve) or SOD1 G93A (n = 6) (lower curve). The addition of SOD1 G93A promoted instability of channel conductance at several voltages applied. At the bottom of the figure, the applied voltage curve.

Purified VDAC1 was reconstituted into a planar phospholipid bilayer (PLB) and its channel conductance was monitored before and after addition of SOD1 proteins, on *cis* or *trans* side of the membrane. Figure 2A shows a typical record of ion current through a single VDAC1 channel, in 1 M KCl and an applied potential of +25 mV. In these experimental conditions, the addition of SOD1 WT did not modify the VDAC1 closed state. Conversely, the addition of SOD1 G93A, on the *cis* side of the membrane, promoted VDAC1 channel instability: the conductance switched from the stable low-conductance state to several high-conducting states, indicating a specific interaction between the two proteins. Moreover, the effect of SOD1 proteins on the voltage-dependence of VDAC1 was monitored by triangular voltage ramps from 0 to ±50 mV. The upper curve in Fig. 2C shows the typical VDAC1 voltage response. The current linearly follows the voltage applied up to about ±25 mV, where VDAC1 decreases its channel conductance through step-like transitions, remaining in low-conducting states. A perturbation of voltage-dependence is exclusively visible when SOD1 G93A interacted with VDAC1 (lower curve, Fig. 2C), while no modification in the usual VDAC1 pattern was noticeable upon addition of SOD1 WT (middle curve, Fig. 2C). In conclusion, VDAC1 loses its ability to linearly respond to the voltages applied in the presence of the mutant SOD1 G93A.

### SOD1 G93A competes with Hexokinase I (HK1) towards binding-site(s) on VDAC1

**Figure 3:**
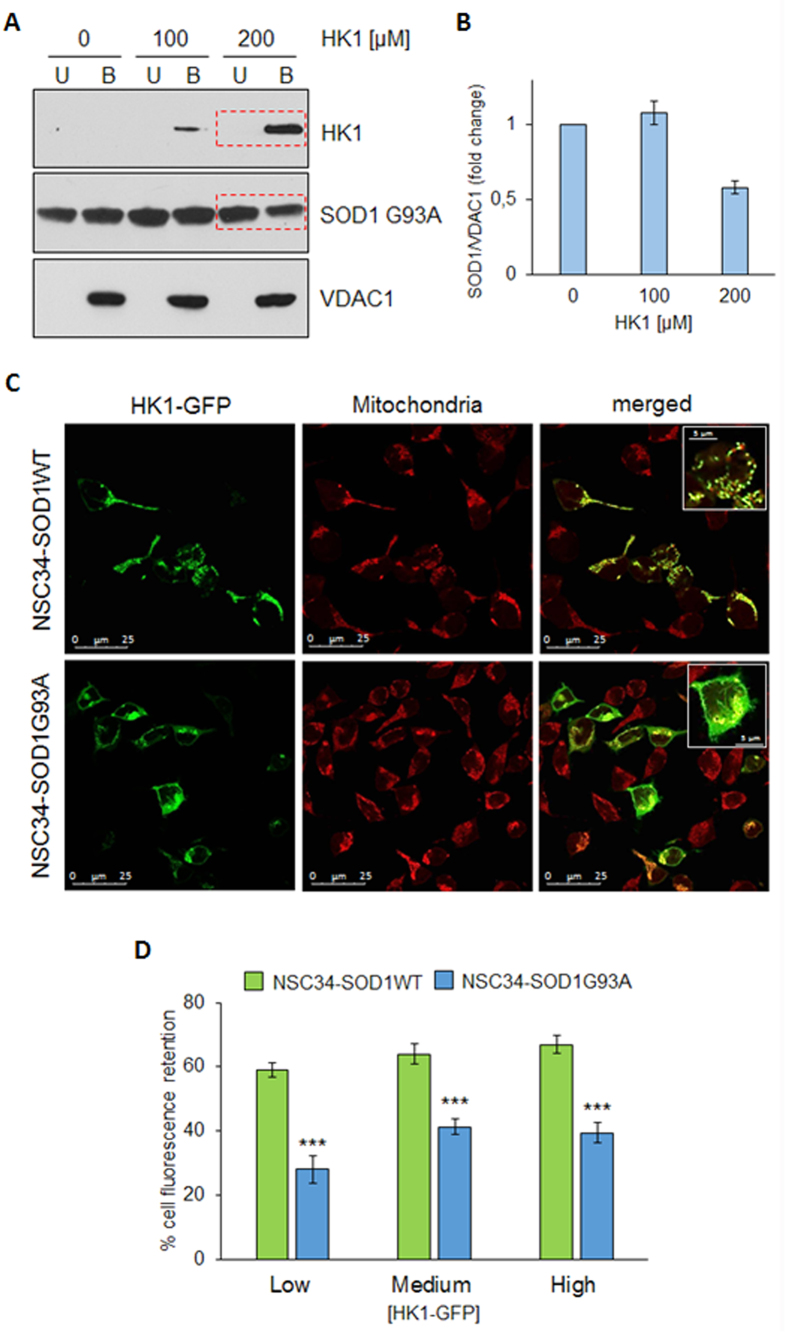
HK1 competes with SOD1 G93A in VDAC1 binding. (**A**) Western blot analysis of the binding between VDAC1 and SOD1 G93A in the presence of increasing concentration of recombinant HK1 (0–200 μM). Affinity of SOD1 G93A for VDAC1 was reduced of about 40% with respect to the control in the presence of 200 μM HK1 (a representative blot from three independent experiments). (**B**) Relative quantification of SOD1 G93A in VDAC1-bound fraction obtained by densitometry. Data are showed as means ± SEM of n = 3. (**C**) Subcellular distribution of HK1-GFP detected by fluorescence microscopy in NSC34-SOD1WT or NSC34-SOD1G93A cells. In presence of SOD1 WT, HK1-GFP almost completely binds mitochondria, as showed by its co-localization with Mito-Tracker; conversely, in the presence of SOD1 G93A, HK1-GFP signal is more diffused. (**D**) HK1-GFP in NSC34 cells is bound to mitochondria and competes with SOD1 G93A. The fluorescent signal of the HK1-GFP was measured after plasma membrane permeabilization by digitonin. The retention of fluorescence indicates that HK1-GFP is associated to intact mitochondria. The fluorescent signal retained in NSC34-SOD1WT or G93A cell was estimated with regard to the increasing amount of HK1-GFP transfected into the cells. Data were normalized to the corresponding controls expressing the same amount of the plasmid carrying the GFP only. The amount of HK1-GFP retention grows as the amount of HK1-GFP plasmid is increased. Lower levels of HK1-GFP retention were found in NSC34-SOD1G93A compared to the SOD1WT. Data are shown as means ± SEM of n = 3; *** < 0,001 compared to NSC34-SOD1WT.

It is well known that HKs bind to VDAC1 in physiological conditions^9^. We hypothesized that the available area for interaction with soluble proteins is a limited, exposed portion of the transmembrane pore VDAC1. This hypothesis suggests that there could be a competition between different proteins towards the same, or close, site(s) of the VDAC1. For this reason, we repeated the VDAC1-SOD1 G93A binding assay, adding increasing concentrations of HK1. We found that SOD1 G93A binds VDAC1 (Fig. 3A): the addition of HK1 strongly decreased (about 40%, Fig. 3B) the SOD1 G93A bound to immobilized-VDAC1 (Fig. 3A), despite the concentration of SOD1 G93A was overwhelming the stoichiometry of VDAC1 binding site(s). The reduction corresponds to the increase of HK1 in VDAC1-bound fraction, and it is proportional to the HK1 concentrations added in the assay (Fig. 3A). Therefore, in such experimental conditions, HK1 is able to impair the VDAC1-SOD1 G93A interaction, suggesting a competition of the two proteins for the same binding site(s).

The interference of HK1 in the VDAC1-SOD1 G93A interaction was further investigated in NSC34 cells stably transfected with inducible human SOD1 G93A (NSC34-SOD1G93A), a recognized ALS cell model, and compared with SOD1 WT (NSC34-SOD1WT) expressing cells^17^. We previously controlled that the motor-neuron NSC34 cell line contain a low level of total HK1 in comparison to other cells, as shown in Fig. S2. HK1 mostly localizes to mitochondria (Fig. S2). NSC34-SOD1G93A or NSC34-SOD1WT cells were then transiently transfected with increasing concentrations of constructs encoding HK1-GFP and the measured fluorescence was related to the mitochondria-bound HK1-GFP. The expression of HK1-GFP was also controlled in NSC34 cells (see Fig. S3), where HK1-GFP localization was also analyzed by fluorescence microscopy. As expected, in NSC34-SOD1WT cells, most of HK1-GFP localized to mitochondria, as demonstrated by the typical punctuated staining (Fig. 3C). However, in NSC34-SOD1G93A cells the HK1-GFP signal became diffused, indicating a partial shift towards the cytosol (Fig. 3C). A quantification of the mitochondria-related fluorescent signal was obtained after a limited permeabilization with digitonin^18^ of NSC34 cells expressing SOD1WT or G93A. In this experiment, the cytosolic GFP fluorescence was allowed to leave the cell and the fluorescence retained inside the cell was assumed to be due to the mitochondria-associated HK1-GFP. Indeed, the permeabilization of NSC34-SOD1WT or G93A stable cell lines, transfected also with GFP, promoted the loss of the signal, in any tested condition. Conversely, the signal was strongly retained (60–70%) in NSC34-SOD1WT expressing also HK1-GFP (Fig. 3D), indicating that HK1-GFP binds to mitochondria, in proportion to the added concentration. Interestingly, in NSC34-SOD1G93A, HK1-GFP in the cell was reduced (30–40% of the control). Therefore, the HK1-GFP ability to bind mitochondria was clearly reduced in the presence of SOD1 G93A, but not of SOD1 WT (Fig. 3D). Considering that HK1 binds mitochondria exclusively docking VDAC1, this result supports our hypothesis that HK1 and SOD1 G93A compete for the same VDAC1 binding site/s.

### A N-terminal HK1-based peptide interacts with VDAC1 and modulates its channel conductance

It is well known that the N-terminal end of HK1 is responsible of the enzyme interaction with VDAC1 and modulates its channel activity^19^,^20^,^21^. To evaluate the ability of HK1 N-terminus to interfere with the binding between VDAC1-SOD1 G93A, we produced a synthetic NHK1 peptide, corresponding to the first 11 amino acids of human HK1.

The electrophysiological behavior of human VDAC1, reconstituted into a PLB, was monitored before and after addition of NHK1 on the *cis* or *trans* side of the membrane.

**Figure 4:**
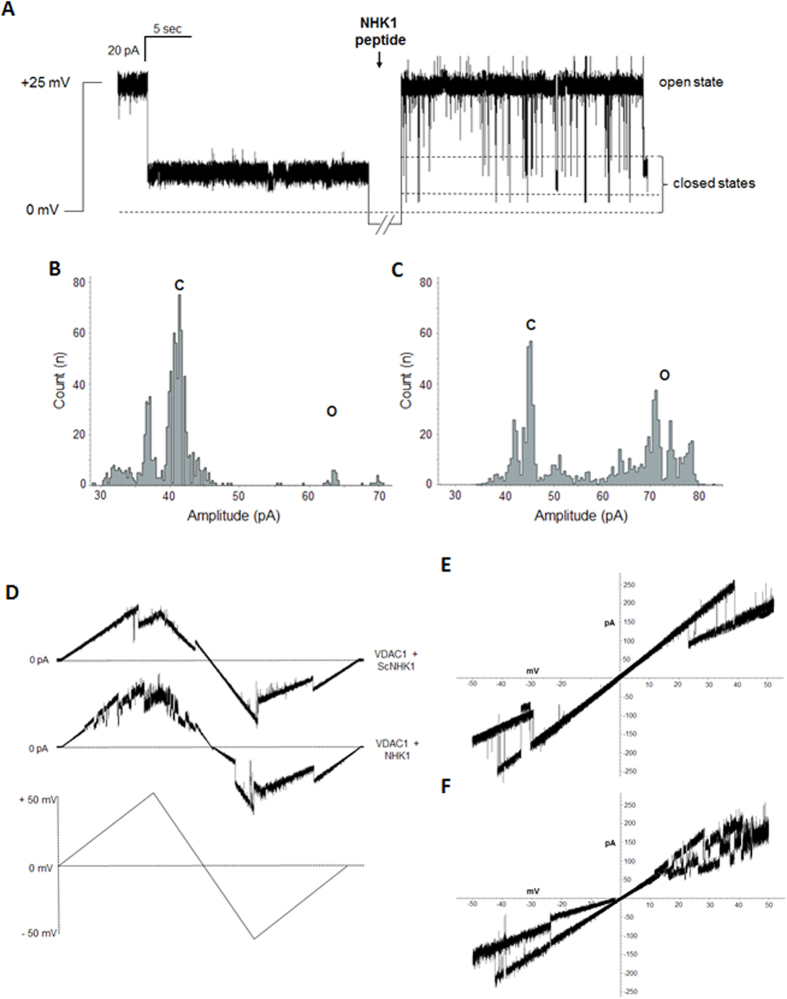
Electrophysiological characterization of the interaction between the NHK1 peptide and VDAC1. (**A**) Single channel analysis showing a representative trace of VDAC1 conductance (n = 5) at +25 mV in 1 M KCl before and after the addition of NHK1 peptide. VDAC1 stably switched to the closed state depending on the applied voltage. Conversely, the addition of NHK1 peptide promoted the instability of the channel, affecting the pore’s gating. (**B**,**C**) Plots of the distribution of channel amplitude events calculated in the absence (**B**) or in the presence of NHK1 peptide (**C**). Amplitude values for VDAC1 alone appeared as a main peak corresponding to the closed state. On the contrary, the presence of NHK1 changed the amplitude values distribution, yielding two distinct peaks, the former corresponding to the closed state and the latter to the open state. (**D**) Triangular curve of different experiments performed in a symmetric 1 M KCl solution with VDAC1 inserted in the membrane upon addition of a scramble peptide (n = 5) (upper curve) or NHK1 peptide (n = 8) (lower curve). The addition of NHK1 peptide promoted the instability of channel conductance at several voltages applied. At the bottom of the figure, the applied voltage curve. (**E,F**) Current-voltage (I–V) curves obtained from the experiments in (**D**). (**E**) I–V plot of VDAC1 alone is a linear trace at low applied voltages (from ±10 to ±25 mV) and show characteristic current transitions at high voltages. (**F**) Conversely, upon NHK1 incubation, VDAC1 I–V trace is extremely noisy (especially at positive voltages), and shows different sub-conductance states.

Under standard experimental condition (1 M KCl and an applied potential of +25 mV) the VDAC1 channel was mainly in low-conducting closed states. The addition of NHK1 on one side of the membrane induced several fast and reversible events of VDAC1 low-conducting closed states (reported in Fig. 4A). The effect of NHK1 on the channel conductance is even more evident by plotting the amplitude values obtained in the current traces as a function of the number of events. While for VDAC1 alone the amplitude values appear as a main peak corresponding to the closed states (Fig. 4B), the presence of NHK1 caused a different distribution of events: an additional, different peak corresponding to the open state was visible, together with the closed states peak (Fig. 4C). No influence on the electrophysiological activity was instead detected upon addition of a scramble peptide (ScNHK1) used as control (data not shown). The effect of NHK1 on the voltage-dependence of VDAC1 was monitored by triangular voltage ramps. Again, while no difference was found in presence of ScNHK1 (upper curve, Fig. 4D), the presence of NHK1 strongly affected the channel voltage-dependence of VDAC1 (lower curve, Fig. 4D). In this experiment, indeed, VDAC1 completely loose, very precociously (already at ±15 mV applied), its ability to linearly respond to the applied voltage: a continuous switch from open to closed states was observed, especially at positive potentials. Similarly, the current vs. voltage (I–V) plot shows that in the presence of only VDAC1, I–V plot is linear in the voltage range ±10–25 mV, and the current transitions (slope), corresponding to decreased conductance states, appeared outside this range (Fig. 4E). Upon NHK1 incubation, VDAC1 current transitions at positive voltages are very noisy and pass through different sub-conductance states (Fig. 4F). Notably, at negative membrane potentials, regions of decreased slope appeared already at −10 mV, suggesting that NHK1 peptide raises VDAC1 sensitivity to negative voltage applied. No similar result, instead, was observed in the presence of ScNHK1 (data not shown).

The electrophysiological analysis strongly indicates the ability of NHK1 peptide to interact specifically with VDAC1 and to modulate its channel conductance and voltage-dependence.

### NHKI peptide highly impairs the interaction between SOD1 G93A and VDAC1

**Figure 5:**
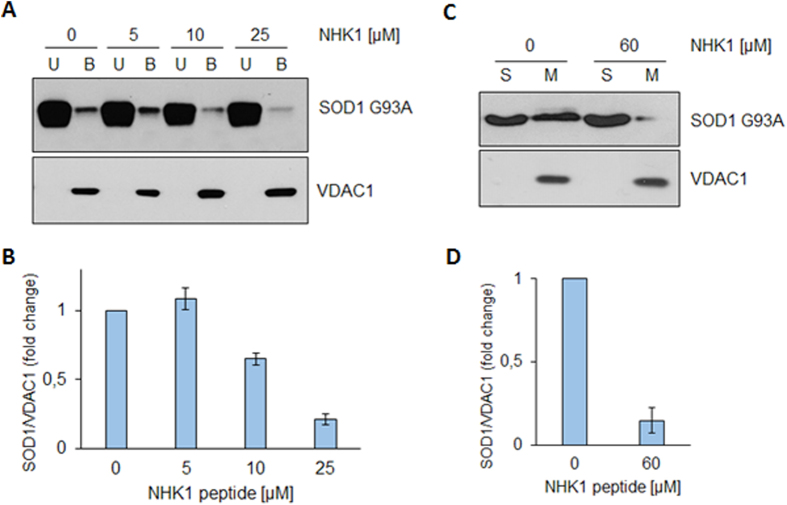
NHK1 peptide impairs VDAC1-SOD1 G93A interaction. (**A**) Representative western blot analysis of three independent experiments of the binding assay between VDAC1 and SOD1 G93A in the presence of increasing concentrations of NHK1 peptide (0–25 μM). NHK1 reduce the binding of SOD1 G93A to VDAC1 *in vitro*. (**B**) Relative quantification of SOD1 G93A in VDAC1-bound fraction obtained by densitometry. Data are showed as means ± SEM of n = 3. (**C**) Representative western blot analysis of three independent experiments of the binding assay between mitochondria and SOD1 G93A in the presence of NHK1 peptide (60 μM). NHK1 dramatically reduces SOD1 G93A in mitochondrial pellet (*M*) to about 80% of control. *S* indicates supernatant fraction containing exceeding SOD1. (**D**) Relative quantification of SOD1 G93A in mitochondrial pellet. Data in (**D**) are means ± SEM of n = 3.

In order to evaluate the NHK1 ability to interfere with VDAC1-SOD1 G93A interaction, a binding assay was performed. Increasing concentrations of NHK1 were added to immobilized-VDAC1, before SOD1 G93A addition. Results in Fig. 5A clearly show that, in the presence of 10 and 25 μM of NHK1 peptide, the amount of SOD1 G93A in VDAC1-bound fraction was reduced by the 40% and 80%, respectively (Fig. 5B). No similar effect was observed by repeating the assay in presence of ScNHK1. This result indicates that, *in vitro*, NHK1 strongly impairs the interaction between VDAC1 and SOD1 G93A in a more effective way than the whole HK1 protein. In fact, a 20-fold smaller concentration of peptide was used to obtain a result similar to that obtained by using the whole HK1 in the previous experiment (see Fig. 3A).

The ability of NHK1 to inhibit SOD1 G93A from binding the mitochondrial surface was investigated using intact mitochondria purified from NSC34 cells. Mitochondria were incubated with SOD1 G93A, in the presence or absence of 60 μM NHK1 peptide. Figure 5C shows that SOD1 G93A was distributed between the supernatant and mitochondrial fractions, in the absence of peptide. Following incubation with NHK1, a dramatic decrease of 85% SOD1 G93A bound to mitochondria was seen (Fig. 5D). Again, no effect was found by incubating mitochondria with the ScNHK1 peptide.

These results prove that NHK1 peptide strongly hinders the SOD1 G93A binding to purified VDAC1 or mitochondria.

### NHK1 peptide restores the NSC34-SOD1G93A cells viability counteracting the mitochondrial dysfunction

**Figure 6:**
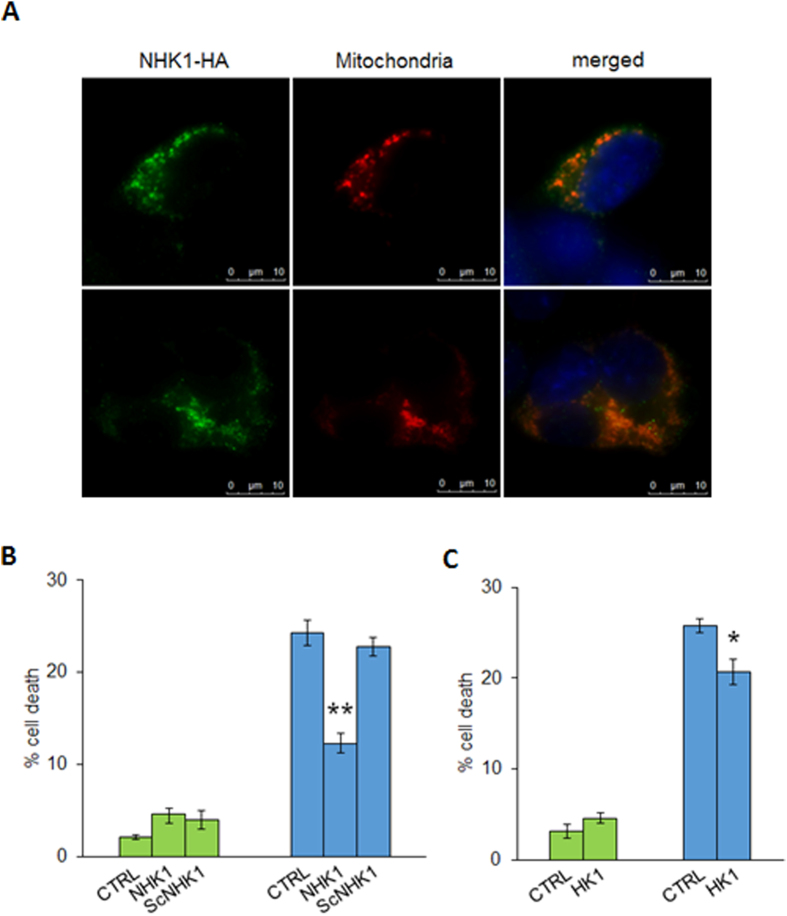
NHK1 peptide localize to mitochondria and improves the cell viability of NSC34 cells. (**A**) Fluorescence microscopy analysis of subcellular distribution of NHK1-HA peptide in NSC34 cells by indirect immunofluorescence targeting the HA tag. Mitochondria were visualized by expressing the mitochondrial-targeted mtDsRED protein. NHK1-HA peptide co-localized with mitochondria. (**B**) Analysis of cell death in NSC34-SOD1WT and G93A analyzed upon transfection with an empty vector (CTRL) or a plasmid encoding either for the NHK1 peptide (NHK1) or the scramble peptide (ScNHK1). Expression of SOD1 G93A produced 20% of NSC34 cell death compared with the same cells expressing SOD1 WT. Conversely, an improvement of cell viability in NSC34-SOD1G93A was observed upon expression of NHK1 peptide. The scramble peptide does not change the cell viability, indicating a specific effect of NHK1 peptide. Data were normalized with the not induced transfected cell. Data are expressed as means ± SEM (n = 3), **p < 0.01 compared to NSC34-SOD1G93A. (**C**) Analysis of NSC34-SOD1WT and G93A cell death upon transfection with empty plasmid (CTRL) or plasmid encoding for HK1. The expression of HK1-GFP in combination to the SOD1 G93A promoted a slight but significant improvement of cell viability. Data were normalized for the correspondent, not induced transfected cell. Data are expressed as means ± SEM (n = 4), *p < 0.05 compared to NSC-34-SOD1G93A CTRL.

In order to analyze the effect of NHK1 peptide in our ALS-like *in cellulo* system, the subcellular distribution of NHK1 peptide was investigated. NSC34 cells were transiently transfected with a plasmid encoding for a HA-tagged NHK1 peptide and for a mitochondrial-targeted Red Fluorescent Protein (mtDsRED)^18^. Immunofluorescence assays in Fig. 6A revealed that NHK1 peptide mainly co-localized with mtDsRED signal, indicating its ability to bind mitochondria in NSC34 cells. Then, the NHK1 peptide, as well as the whole HK1 as control, was expressed in NSC34-SOD1G93A or SOD1WT to evaluate its ability to counteract the toxicity mediated by mutSOD1. Literature reports indicate that SOD1 mutants expression in NSC34 cell promote a significative loss of cell viability, accordingly with the specific mutation^22^. Our results show that, upon expression of SOD1 G93A, NSC34 cells loose about 20% of cell survival (Fig. 6B). However, this toxic effect was counteracted by the NHK1 expression: an improvement of cell viability, (about 50% of the control (Fig. 6B)) was observed; furthermore, a similar but lower effect was also found upon overexpression of the whole HK1 (Fig. 6C). Therefore, supplementation of NHK1 peptide (or HK1) to NSC34-SOD1G93A cells promotes a recovery of the cell viability.

**Figure 7:**
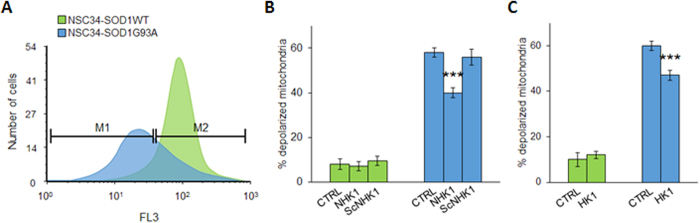
NHK1 peptide recovers the mitochondrial functionality of NSC34 cells. (**A**) Representative Flow cytometry 1D plots of induced NSC34-SOD1WT or G93A clones stained with TMRM. The fluorescence was read on FL3 channel (λex 550 nm/λem 590 nm). Peak corresponding to NSC34-SOD1G93A is shifted toward lower values of fluorescence. M1 indicates cells with low ΔΨm values, M2 indicates cells with high ΔΨm. (**B**) Mitochondrial depolarization rate of NSC34 cells is affected by the presence of NHK1 peptide. Mitochondria depolarization was estimated by TMRM fluorescence in NSC34-SOD1WT or G93A expressing NHK1 or ScNHK1 peptide. A high rate of mitochondrial depolarization was found for NSC34-SOD1G93A, while only few depolarized mitochondria were observed in NSC34-SOD1WT. However, reduction of depolarization was observed upon expression of NHK1 peptide, but not ScNHK1. Data were normalized with the correspondent, not induced cells. Gates reported in (**A**) were used for quantification. Data are expressed as means ± SEM (n = 3), ***p < 0.001 compared to NSC34-SOD1G93A CTRL. (**C**) Mitochondrial depolarization of NSC34 cells in the presence of HK1. Mitochondria depolarization was estimated by RH123 fluorescence in NSC34-SOD1WT or G93A expressing HK1. Depolarization was reduced by HK1 expression in NSC34-SOD1G93A. Data were normalized with correspondent not induced cells, and are expressed as means ± SEM (n = 3). ***p < 0.001 compared to NSC34-SOD1G93A CTRL.

To evaluate the NHK1 influence on mitochondrial functionality, mitochondrial membrane potential variation (ΔΨ_m_) was assayed in the presence of NHK1 peptide or HK1. ΔΨ_m_ is related to the ATP production by oxidative phosphorylation; therefore, it is considered an indication of good mitochondrial and cellular health^23^,^24^. Using mitochondria-targeted fluorescent probes, we estimated in flow cytometry the rate of mitochondrial depolarization. NSC34-SOD1G93A cells shown a high level of depolarized mitochondria compared to NSC34-SOD1WT. Indeed, as showed in Fig. 7A, the emission peak of fluorescence for NSC34-SOD1G93A cells was significantly lower compared to the NSC34-SOD1WT peak. The uptake of the probe into mitochondria is ΔΨm-dependent: thus this result means that SOD1 G93A expression strongly affects the cell energetic metabolism. A quantification of fluorescent-negative NSC34-SOD1G93A cells, corresponding to the depolarized mitochondria rate, was performed as previously reported^24^, and resulted in a dramatic increase of about 60%, compared to NSC34-SOD1WT (Fig. 7B). In this dramatic situation, the NHK1 expression, but not ScNHK1, resulted in a partial recovery of the physiological ΔΨm, since depolarized mitochondria were reduced by 20% in NSC34-SOD1G93A cells (Fig. 7B, S4). Similarly to NHK1 peptide, the expression of the whole HK1 reduced the depolarized mitochondria of about 15% (Fig. 7C). In conclusion, the NHK1 peptide, and minimally the whole HK1, is able to partially recover the mitochondrial functionality and, consequently, the cell vitality in the ALS model NSC34 cells.

## Discussion

A previous report showed that mutSOD1 interact with VDAC1 in spinal cord mitochondria from ALS model rat^6^. In our work we preliminary confirmed that the mutant SOD1 G93A, but not the SOD1 WT, is able to interact with VDAC1 immobilized on magnetic beads. Furthermore, we determined, by MST analysis, the binding affinity of SOD1 G93A with VDAC1 and the effect of SOD1 G93A on VDAC1 conductance. Our electrophysiological data showed that, at the voltages applied which stably close VDAC1, SOD1 G93A, but not the SOD1 WT, promotes a prolonged instability of VDAC1 conductance. On the other side, Israelson and coworkers demonstated that addition of SOD1 G93A to PLB-reconstituted VDAC1, at the voltages applied which stably maintain VDAC1 in an open state, promotes a partial closure of VDAC1 channels^6^. Therefore, both results strongly support a direct effect of mutated SOD1 G93A on VDAC1 conductance. From the literature and from these convincing results we hypothesized that VDAC1 could be the specific docking site on the OMM for the SOD1 G93A, and possibly for all SOD1 mutants. The influence of the interaction in the gating features of VDAC1 could indeed explain the mechanism of impairment of the bioenergetics metabolism and the oxidative stress of the ALS MNs^6^. In this perspective, the physiological interactions involving VDAC1 in spinal cord MNs could be altered in ALS, giving thus an explanation for the specific susceptibility to the disease showed by this tissue.

Another recent report showed that SOD1 G93A, but unexpectedly also SOD1 WT, preferentially bound to Bcl2, rather than VDAC1, in ALS mitochondria^13^. The binding promoted a conformational change of Bcl2 that, in turn, altered its physiological interaction with VDAC1, producing mitochondrial dysfunction^13^. This means that SOD1 G93A might bind VDAC1 or alternatively Bcl2. However, a detailed analysis of gene expression in mouse spinal cords reveals that Bcl2 level is extremely low and further decreases with the aging of mice^25^. In the same database, it is shown that VDAC1 is highly expressed in brain and spinal cord with no special age difference^25^. Therefore, the VDAC1-SOD1 G93A interaction should be predominant in ALS spinal cord MNs, in comparison to VDAC1-Bcl2 interaction. This is further strengthen by the ability of specific antibodies against misfolded SOD1 proteins (e.g. against DES2-3H1 SOD1 domain) to recognize SOD1 mutant deposits interacting with VDAC1 on the cytosolic surface of mitochondria^26^. Moreover, it has also been clearly reported that mitochondria from brain or spinal cord differentially respond to injury, with an imbalance in oxidative stress that may contribute to the susceptibility of spinal cord MNs in neuropathologies^27^. Considering these evidences, we have addressed our work towards the characterization of the binding site of mutSOD1 on the surface of ALS MNs mitochondria.

VDAC1, the main pore and the most abundant protein of the OMM, is also known as the receptor of HK1, an important enzyme for the energy production^11^. HK1 exploits the VDAC1 as a channel bringing the newly synthesized ATP to glucose phosphorylation. HK1, as HK2, utilizing the N-terminal domain, binds to VDAC1. The interaction with HK1 has also another important consequence: it can modulate the VDAC1 activity^20^ and the Bcl2-VDAC1 interaction, which in turn, regulates the apoptotic intrinsic pathway^10^. Intriguingly, brain is very rich of HK1 and HK2, while spinal cord shows a peculiarly low HK1 concentration and no HK2 expression^6^,^25^,^28^. It is known that, in affected tissues of ALS rat, VDAC1-linked mutSOD1 level is inversely correlated to the HK1 concentration^6^. We thus hypothesize that in spinal cord mitochondria from ALS MNs, a competition for the VDAC1 binding site might happen between mutSOD1 and HK1, the physiological interactor. When the mutSOD1 affinity, or concentration, is higher than HK1, mitochondria can be subject to a double effect: to become more susceptible to the mutSOD1-mediated toxicity and to lose the anti-apoptotic protection exerted by HK1 when bound to the organelle.

In this work, by using *in vitro* and *in cellulo* approaches, we proved that HK1 and in particular its N-terminal domain, competes with SOD1 G93A for binding to common site/s on VDAC1. The *in vitro* results show that the increase of HK1 concentration promotes a decrease of SOD1 G93A affinity for the immobilized VDAC1. In addition, we demonstrate that HK1-GFP in NSC34 cells highly competes with SOD1 G93A for binding VDAC1. Conversely, SOD1 WT expression does not compete with HK1 for the organelle’s binding site. We also demonstrated that HK1-GFP counteracts the mitochondria toxicity found in NSC34-SOD1G93A. Increasing evidences indicate that HKs play a decisive role not only in glycolysis but also in cell survival, carrying out a defence action on many cell types or contrasting disease pathways^29^,^30^,^31^,^32^. Not coincidentally, VDAC1-HKs complexes allow the cancer cells to grow faster (Warburg effect) and, to date, destroying these complexes has been considered a putative therapy^33^,^34^.

In order to develop our findings towards a perspective therapy for ALS, we produced a synthetic NHK1 peptide corresponding to the N-terminal domain of human HK1, and tested it in our experimental models. Our interest in this domain comes from the consideration that it is sufficient to specifically target the enzyme to VDAC1, as demonstrated when it is deleted from the protein^19^,^21^,^35^. Moreover, the moiety corresponding to the first 11 residues of HK1 represents the most hydrophobic part of N-terminus, forming a “tail” structured in α-helix, which is believed responsible to take contact with the OMM and, thus, with VDAC1^36^,^37^. We have found that the NHK1 peptide specifically interacts with VDAC1, destabilizing its gating properties and considerably modifying its conductance at any voltage applied. Moreover, NHK1 peptide highly counteracts the SOD1 G93A binding to beads-immobilized VDAC1, about 20 times more efficiently than the whole HK1. Similar results were found also in isolated mitochondria and in the chosen ALS cell model (NSC34-SOD1G93A). Interestingly, the expression of NHK1 peptide in NSC34-SOD1G93A produced an important decrease in the toxicity mediated by mutSOD1, and promoted the recovery of mitochondrial depolarization. In conclusion, the NHK1 peptide in our hands contrasts the VDAC1 interaction with SOD1 G93A. As a consequence, the measured life parameters remarkably improved.

Although in this work we support the VDAC1 involvement in ALS mitochondrial dysfunction, we are well aware that many other aspects remain to be clarified. To give just an example, the amino acid sequence of the three VDAC isoforms^38^ is conserved, thus it is possible that the commercially available antibodies against VDAC do not perfectly match the VDAC1 isoform. While VDAC2 does not seem to interact with mutSOD1^6^, no information about VDAC3 involvement is available, even if this isoform is expressed in the spinal cord as much as VDAC1 and it was shown that it is able to interact with a number of cytosolic proteins^39^. Therefore, it will also be interesting to examine the possible involvement of VDAC3 in ALS, especially in light of its ability to function as a buffer for mitochondrial ROS^40^. Furthermore, mitochondrial dysfunction and aggregation of toxic peptides/proteins correlate not only to ALS, but also to other neurodegenerative diseases, like Alzheimer’s^41^ or Parkinson’s^42^. Interestingly, amyloid beta peptide, such as alpha-synuclein, bind VDAC1^43^,^44^. Consequently, we suppose that NHK1 peptide could contrast also these specific interactions, producing a healthy effect in Alzheimer or Parkinson disease.

**Figure 8:**
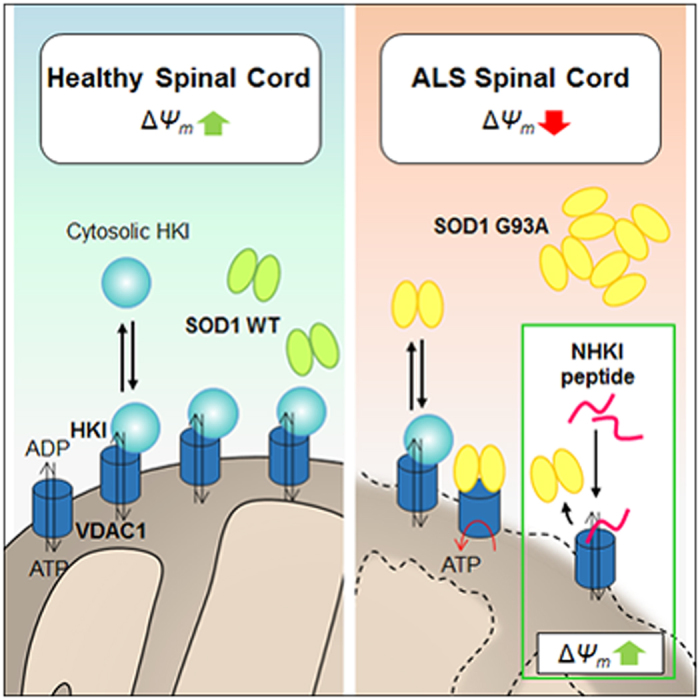
A proposed mechanism of action of NHK1 peptide. In physiological conditions, VDAC1 is the receptor of HK1, but not of SOD1 WT. Conversely, in ALS, SOD1 G93A binds VDAC1 and impairs HK1 binding. As a consequence, VDAC1 channel conductance and metabolic exchanges through VDAC1 are strongly impaired, thus promoting mitochondrial dysfunction. NHK1 peptide inhibits SOD1 G93A binding to VDAC1 and ameliorates mitochondrial dysfunction and, in general, the cell viability.

In conclusion, our results provide new insight into the mechanism underlying the benefits of mutSOD1/VDAC1 inhibition against neurodegeneration and give details to explain the aetiology of ALS. In addition, we propose that NHK1 peptide, possibly mimicking the contact surfaces between SOD1 G93A and VDAC1, can be used to interfere with this interaction and relieve the ALS mitochondrial dysfunction recovering cell viability (Fig. 8). Our work suggests for NHK1 peptide a powerful neuroprotective potential which needs to be especially assessed for its therapeutic relevance in ALS.

## Methods

### Preparation of recombinant proteins and peptide

The N-terminal 6xHis-tagged VDAC1, the Strep-tagged SOD1 WT and the ALS-linked SOD1 G93A mutant were expressed in *E. coli* and purified as detailed explained in Supplementary Information. The recombinant human HK1 was purchased by Sigma.

A peptide corresponding to residues 2–12 (N-terminal region) of human HK1 protein (IAAQLLAYYFT) was produced by GenScript Inc. (Piscataway, USA) and is called in the text: NHK1 peptide. A scramble peptide, ScNHK1 (FAQLTIALAYY), was synthetized and used as control.

### Mitochondrial binding assay

Intact mitochondria were purified from NSC34 cells by Mitochondrial isolation kit (Miltenyi Biotech), according with manufacturer’s instructions. Isolated, intact mitochondria, in the storage buffer provided, were quantified by Lowry method. Mitochondria were incubated with 2 μg of purified SOD1 WT or G93A for 1 h at RT, under constant shaking in storage buffer. Alternatively, a pre-incubation with 60 μM NHK1 peptide for 1 h, in the same condition, was performed. Mitochondrial pellet was collected by centrifugation for 2 min at 13000×g. Supernatants were collected, and mitochondrial pellets were lysed using 3% Triton X-100. Proteins in mitochondrial or supernatant fraction were detected by western blot and quantified as explained in Supplementary Information. At least, three independent experiments were performed.

### *In vitro* VDAC1-SOD1 binding assays

Recombinant VDAC1 was immobilized onto Ni-NTA Magnetic Agarose Beads (Qiagen) by exploiting its 6xHis-tag, according with manufacture’s protocol. For each experiment, a large excess of VDAC1 (30 μM) were incubated with 50 μL of beads suspension in Interaction Buffer (IB) (300 mM NaCl, 50 mM NaH_2_PO_4_, 20 mM imidazole, pH 8.0) to saturate all sites. Beads were collected using the magnet-based system DynaMag-2 (Life Technologies) and washed twice in Wash Buffer (300 mM NaCl, 50 mM NaH_2_PO_4_, 20 mM imidazole, 0.05% Tween-20, pH 6.3) to remove any excess of VDAC1. Beads-immobilized VDAC1 were incubated with 6.25 μM SOD1 WT or G93A, for 1 h at RT, under constant shaking, in 100 μL of IB. The assay was also performed by pre-incubating immobilized-VDAC1 for an extra 1 h with increasing concentration of purified human HK1 (0, 100, 200 μM) or NHK1 peptide (0, 5, 10, 25 μM), before SOD1 G93A addition. Beads were recovered using Dyna-Mag2 system and VDAC1-complexes were eluted from beads using 50 μL of Elution Buffer (300 mM NaCl, 50 mM NaH_2_PO_4_, 300 mM imidazole, pH 3.5). Any protein in VDAC1-bound and -unbound fractions was evaluated by western blot. Three independent experiments were performed for each condition tested.

### Microscale Thermophoresis

Microscale Thermophoresis (MST) analysis was performed using the NanoTemper Monolith NT.115 apparatus (NanoTemper Technologies, Munich, Germany) as described^45^. VDAC1 was fluorescently-labeled using the NanoTemper protein-labeling kit Green/Blue. 100 nM labeled-VDAC1 were incubated, for 5 min at RT in the dark, with serial dilutions of SOD1 WT or G93A (from 1.22 nM to 20 μM), in PBS with 0.05% Tween 20. Afterwards, the samples were loaded into a glass capillary (Monolith NT Capillaries) and thermophoresis analysis was performed (light-emitting diode 20%, IR laser 20 to 80%). In every determination at least three independent experiments were performed.

### VDAC1 channel reconstitution, recording and analysis

Purified VDAC1 was reconstituted into a Planar Lipid Bilayer (PLB) apparatus (Warner Instruments, Hamden, CT, USA), previously described^46^. Bilayers were prepared using asolectin (Sigma) dissolved in decane (Sigma) containing 1% chloroform (Sigma) across a 200 μm hole in a derlin cuvette (Warner Instruments). Experiments were performed in 1 M KCl, 10 mM Hepes, pH 7.0^40^,^46^. Control experiments using empty membrane and/or detergents were performed to avoid activity in any of the above solutions. Data were acquired using a Bilayer Clamp amplifier (Warner Instruments) at 100 μs/point, filtered at 200 Hz and analyzed offline using Clampfit 10.4 program set (Axon Instruments, Union City, CA, USA). Single channel analysis of VDAC1 was performed in presence or not of 0.2 μM of SOD1 WT or G93A, 15 μM NHK1 or ScNHK1 peptide.

### Motor neuron cell lines

The mouse motor neuron-like NSC34 cell line (CELLutions Biosystem Inc.), and/or the NSC34 cells stably transfected with pTet-ON plasmid (Clontech) harboring sequences encoding for SOD1 WT (NSC34-SOD1WT) or G93A (NSC34-SOD1G93A)^17^ were used as ALS model cell line. Cell maintenance, induction, plasmids and transfection condition are explained in details in Supplementary Information.

### Analysis of HK1-GFP mitochondrial retention signal

NSC34-SOD1WT and NSC34-SOD1G93A cells were seeded on 24-well plates and transfected after 24 h from induction using 0,25 (low), 0,5 (medium) or 1 (high) μg of pEGFP-N1-HK1 or empty pEGFP-N1. Cells were harvested after additional 24 h by trypsinization and split into two separate tubes. One series was resuspended in Krebs Ringer Buffered Saline (130 mM NaCl, 3.6 mM KCl, 10 mM HEPES, 2 mM NaHCO_3_, 0.5 mM NaH_2_PO_4_, 0.5 mM MgCl_2_, 1.5 mM CaCl_2_, 4.5 g/l glucose, pH 7.4) to obtain the not-permeabilized sample. The other series was subject to selective permeabilization of plasma membrane by incubation for 2 mins with 25 μg/mL digitonin (Sigma) in Intracellular Buffer (130 mM KCl, 10 mM NaCl, 20 mM HEPES, 1 mM MgSO_4_, 5 mM succinate pH 7.2) supplemented with 50 μM EGTA to avoid Ca^2+^ induction of the permeability transition in the mitochondria of permeabilized cells. Cells were then analyzed by flow cytometry on FL1 log mode. The retention of HK1-GFP signal was calculated considering the residual GFP signal in the permeabilized sample compared to the respective non permeabilized one. The loss of the GFP signal in cells expressing a cytosolic GFP was used as control. Data reported are representative for three sets of independent experiments, each performed in triplicate and based on 100.000 events for each group. Data were statistical analyzed by chi-square test. A p < 0,001 was taken as significant.

### Cell viability assay

24 h-induced NSC34-SOD1WT and NSC34-SOD1G93A cells seeded on 24-well plates were transfected using 0,25 μg of pEGFP-N1-HK1 or empty pEGFP-N1 or 0,5 μg of pMCS-mtDsRED-NHK1 or scramble or empty pCMS-mtDsRED. Cell viability was analyzed using 3-(4,5-dimethylthiazol-2-yl)-2,5-diphenyltetrazolium bromide (MTT)^47^ (Sigma). A colorimetric assay was performed using the microplate reader Varioskan (Thermo Scientific). Reported data are representative for three sets of independent experiments, each performed in triplicate. Data were statistically analyzed using one-way ANOVA followed by Bonferroni post hoc test. The values p < 0.05 and p < 0.01 were taken as significant.

### Measurements of mitochondrial membrane potential (ΔΨ_m_)

ΔΨ_m_ was measured using tetramethyl-rhodamine methyl ester (TMRM) or Rhodamine 123 (RH123) (Molecular Probes) according to the excitation/emission wavelength of the transfection reporter or of the transfected fluorescent protein used. Both probes accumulates in active mitochondria due to their positive charge, whereby the reduction of ΔΨ_m_ leads to the release of fluorescent probes. Transfected NSC34-SOD1WT and NSC34-SOD1G93A cells were washed with PBS and incubated for 30 min at 37 °C with Krebs Ringer Buffered Saline supplemented with either 200 nM TMRM or 0.1 μg/mL RH123 and 20 μM Verapamil (as a multi drug-resistant pump inhibitor) (Sigma). Cells were then harvested and immediately analyzed by flow cytometry. Cells were excited by an air-cooled argon 488 nm laser, reading the GFP fluorescence of HK1-GFP or RH123 fluorescence in FL1 log mode; TMRM and mtDsRED used as transfection reporter of NHK1 peptide were read on FL3 log mode. Only viable cells detected by reading the scattering indicated as FSC and SSC, were considered for our analysis. The threshold physiological value of ΔΨ_m_ was estimated by using cells exposed to 1 μM of the uncoupling agent FCCP as a negative control. Data reported are representative for three sets of independent experiments, each performed at least in triplicate and based on 20.000 events for each group. Data were statistical analyzed by chi-square test. A p < 0,001 was taken as significant.

### Flow cytometry

A CyFlow^®^ ML flow cytometer (Partec) system was used. The system is equipped with three laser sources and 10 optical parameters with dedicated filter setting and a high numerical aperture microscope objective (50 NA 0.82) for the detection of different scatter and fluorescence signals. Data obtained were acquired and gated by using the FCS Express 4 software (DeNovo).

### Statistical analysis

Significance was determined as reported and indicated as *p < 0.05, **p < 0.01 and ***p < 0.001.

## Additional Information

**How to cite this article**: Magrì, A. *et al*. Hexokinase I N-terminal based peptide prevents the VDAC1-SOD1 G93A interaction and re-establishes ALS cell viability. *Sci. Rep.* **6**, 34802; doi: 10.1038/srep34802 (2016).

## Supplementary Material

Supplementary Information
