# Supplementary material for: Hexokinase I N-terminal based peptide prevents the VDAC1-SOD1 G93A interaction and re-establishes ALS cell viability
Source: Sci Rep. 2016 Oct 10;6:34802. doi: 10.1038/srep34802 (PMC5056396; doi:10.1038/srep34802)
Supplement: Supplementary Information [file srep34802-s1.pdf]

Hexokinase I N-terminal based peptide prevents the VDAC1-SOD1 G93A interaction and reestablishes ALS cell viability

Andrea Magrì[1,2,3], Ramona Belfiore[2,3], Simona Reina[1,2,3], Marianna Flora Tomasello[4], Maria Carmela Di Rosa[1,2], Francesca Guarino[2,3], Loredana Leggio[1,2], Vito De Pinto[2,3], Angela Messina[1,2] *

[1]Department of Biological, Geological and Environmental Sciences, Section of Biochemistry and Molecular Biology, University of Catania, Italy

[2]National Institute of Biostructures and Biosystems (INBB), Italy

[3]Department of Biomedical and Biotechnological Sciences, University of Catania, Italy [4]CNR Institute of Biostructures and Bioimaging, Catania, Italy

*Correspondence to: Angela Messina, Department of Biological, Geological and Environmental Sciences, Section of Biochemistry and Molecular Biology, University of Catania, Italy; email: mess@unict.it

Supplementary Information

Supplementary Methods

Cloning of recombinant proteins

The sequence encoding the human VDAC1 was inserted into the pET-21a expression vector (Novagen) in frame with 6xHis tag at C-terminal domain, as previously reported[1]. The sequence encoding the human SOD1 wild type (SOD1 WT) was amplified by *in stock* plasmid using a specific couple of primers (SOD1 fw and rev) and cloned into the pET-52b expression vector (Novagen) in frame with Strep-tag at its Nterminal domain using SanDI/SacI sites. To generate the construct for the ALS-linked SOD1 G93A, the QuikChange II site-direct mutagenesis kit (Agilent) was used in combination with a specific couple of mutagenic primers (SOD1 mutG93A fw and rev). Primer sequences are listed in Supplementary Table 1. All sequences were verified by sequencing.

Expression, purification and refolding of VDAC1 proteins

E. coli BL21 (DE3) cells were transformed with the pET constructs harboring VDAC1 sequences. The 6xHis-tagged VDAC1 proteins were induced, purified and refolded as previously reported[1]. The protein purity was verified in 12% SDS-PAGE followed by Comassie staining. Purified samples were stored at -20°C until further use.

Expression, purification and activity assay of SOD1 proteins

The pET vectors containing SOD1 constructs were used for *E. coli* BL21 (DE3) transformation. Protein expression was achieved by 1 mM isopropyl-β-D-thiogalactopyranoside (IPTG) (Sigma) induction at

18°C over night. Cell lysis and purification of Strep-tagged proteins by affinity chromatography using Strep-Tactin Superflow Plus Resin (Qiagen) were performed under native conditions, according to manufacturer's protocol. Metal loading and storage was performed as reported[2]. The protein purity was verified by 12% SDS-PAGE and Comassie staining. Activity of SOD1 proteins was assayed *in gel* with the Riboflavin/NitroBlue Tetrazolium (RF/NBT) assay as reported[3].

Western blot analysis

Protein fractions were separated by SDS-PAGE using 4-12% Bis-Tris NuPage gel (Invitrogen) and electro-transferred to nitrocellulose membrane for Western blot analysis. The following antibodies were used for immunoblotting: SOD1 (C-17) antibody (1:1000) (Santa Cruz Biotechnology, Inc), VDAC1/porin antibody (1:1000) (Abcam), HKI antibody (1:500) (Abcam), Actin antibody (1:1000) (Sigma). A relative quantification was performed by densitometry analysis using Image Studio Lite software (LI-COR Biosciences), using VDAC1 or actin as loading control.

Motor neuron cell lines maintenance

NSC-34 cells were cultured in 5% CO_2 in DMEM (Gibco) supplemented with 10% fetal bovine serum (FBS) (Gibco) and 1% penicillin/streptomycin (P/S). NSC-34-SOD1WT and NSC-34-SOD1G93A cells were cultured in 5% CO_2 in DMEM F12 (Euro Clone), 10% of tetracycline-free FBS (Euro Clone), 1% P/S, in the presence of 200 µg/mL G418 (Carlo Erba) for selection maintenance. Induction of SOD1 proteins was obtained by addition of 2 µg/mL tetracycline to the medium, as described[4]. Analysis were performed after 48h from induction.

Plasmids and cell transfection

Sequence encoding for human HK1 was amplified from *in stock* plasmid using a specific couple of primers (HK1 fw and rev, see Supporting Table 1 for sequence) and cloned into mammalian expression vector pEGFP-N1 in frame with eGFP sequence at C-terminal domain. The sequence encoding for 2-12 amino acid sequence of HK1were cloned by NheI/SalI digestion into the modified mammalian expression vector pCMS-mtDsRED[5]. pCMS-mtDsRED encodes for the Red Fluorescent Protein of *Discosoma sp.* targeted to mitochondria, used also as transfection marker. Oligonucleotides encoding for NHK1 peptide (NHK1 oligo fw and rev) or for scramble ScNHK1 peptide (ScNHK1 oligo fw and rev) were subject to annealing in order to obtain double strand DNA. Additional couples of oligonucleotides lacking stop codon were cloned in pCMS-mtDsRED with the HA-tag at the C-terminal domain. Oligonulceotides sequences are listed in Supplementary Table 2. All sequences were verified by sequencing. Cells were transiently transfected using Lipofectamine 3000 (Invitrogen) according with the manufacturer's instructions.

Indirect immunofluorescence of adherent cells

NSC34 cells not transfected or transfected with 0,5 µg of pCMS-mtDsRED-NHK1-HA were fixed in 3.7% formaldehyde and permeabilized using 0.3% Triton X-100. Unspecific binding was blocked by 30 min of incubation in 0,2 % gelatin in PBS. Endogenous HK1 was detected in not transfected cells by incubating over-night cells with rabbit anti-HK1 antibody (1:100) (Abcam). NHK1-HA was detected in transfected cells by incubating over-night cells with rabbit anti-HA antibody (1:100) (Santa Cruz Biotechnology, Inc.). After PBS washing, cells were exposed for 1h at RT to the secondary anti-rabbit antibody AlexaFluor 488. Coverslips were mounted with the ProLong Gold antifade mounting medium (Invitrogen) and

examined by fluorescence microscopy. Co-localization with mitochondria was obtained by merging the signal HK1 or HA respectively with the signal from Mito-Traker Red (Molecular Probes) or the fluorescent reporter mtDsRED.

Fluorescence microscopy

A Leica DMI 6000B epifluorescence inverted microscope with Adaptive Focus Control was used. This system is outfitted with a controllable X-cite mercury lamp and an extensive collection of filter cubes (360, 488, 560, 604 nm excitation) for fluorescent microscopy, and a halogen lamp for bright field and DIC. It is equipped with 4 bright lenses (10, 20, 40, 63x), a high resolution Hamamatsu Orca R2 CCD camera (1344x1024 pixels), and motorized stage (XY only). Images were obtained by using the Leica LAS Extended Annotation software.

Supplementary Tables

**SOD1 fw**	5′-TTTGGGACCCATGGCCACGAAGGCCGTGTGCGTG-3′
**SOD1 rev**	5′-TTTGAGCTCTTATTGGGCGATCCCAATTACACC-3′
**SOD1 mutG93A fw**	5′-ACTGCTGACAAAGATG<u>CTG</u>TGGCCGATGTGTCT-3′
**SOD1 mutG93A rev**	5′-AGACACATCGGCCACAGCATCTTTGTCAGCAGT-3′
**HK1 fw**	5′-TTTTGCTAGCATGATCGCCGCGCAGCTCCT-3′
**HK1 rev**	5′-TTTTGTCGACTTAGCTGCTTGCCTCTGTGCGTAA-3′

Table S1. ***List of the primer sequences used in this work.***

**NHK1 oligo fw**	5′-CTAGCATGATCGCCGCGCAGCTCCTGGCCTATTACTTCACGTGAG-3′
**NHK1 oligo rev**	5′-TCGACTCACGTGAAGTAATAGGCCAGGAGCTGCGCGGCGATCATG-3'
**ScNHK1 oligo fw**	5′-CTAGCATGTTCGCCCAGCTCACGATCGCCCTGGCGTATTACTAAG-3′
**ScNHK1 oligo rev**	5′-TCGACTTAGTAATACGCCAGGGCGATCGTGAGCTGGGCGAACATG-3′

Table S2. ***List of oligonucleotides sequences used in this work.***

Supplementary Figures

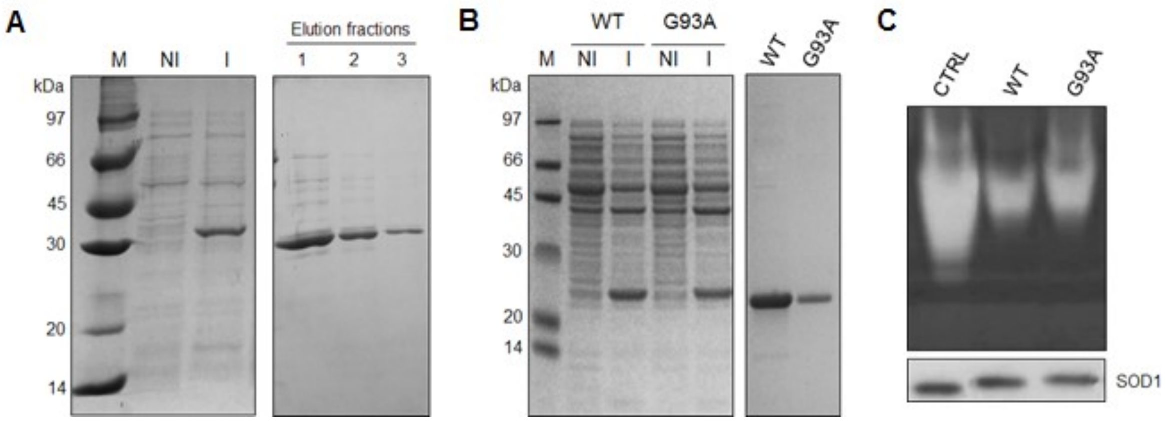

Figure S1. ***Production of recombinant proteins used in this work.***

(A) Electrophoretic analysis of total lysate of *E. coli* BL21 strain transformed with pET construct encoding for the 6xHis-tagged VDAC1 and electrophoretic analysis of eluted fractions after purification. As shown, a clear band of around 32 kDa is visible in IPTG-induced (I), but not in not induced (NI) sample. *M*: molecular weight marker.

(B) Electrophoretic analysis of total lysate of *E. coli* BL21 strain transformed with pET construct encoding for the Strep-tagged SOD1wt and G93A and electrophoretic analysis of eluted fractions after purification. As shown, a clear band appeared at the expected molecular weight in IPTG-induced (I), but not in not induced (NI) sample. *M*: molecular weight markers.

(C) In-gel SOD1 activity assay and relative Western blot analysis. Recombinant SOD1wt and G93A were active. As a control, a SOD1 purchased from Sigma was used (CTRL).

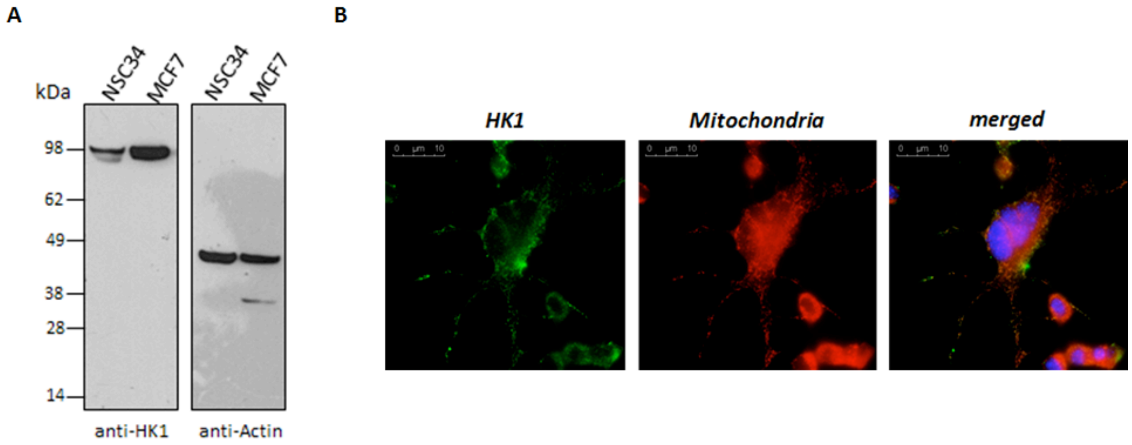

Figure S2. ***Analysis of endogenous HK1 expression in NSC34 cells.***

(A) Western blot analysis of HK1 expression in total lysate of NSC34 or MCF7 cells. MCF7 cells were used here as control, since HK1 is abundant in this cell line. As reported, total level of HK1 is significantly lower in NSC34 cells compared to that of control. Actin was used as loading control.

(B) Indirect immunofluorescence of adherent NSC34 cells with anti-HK1 antibody shows the subcellular distribution of endogenous HK1. As showed by the typical punctuated deposit and by the co-localization with Mito-Tracker, most of HK1 localized to mitochondria.

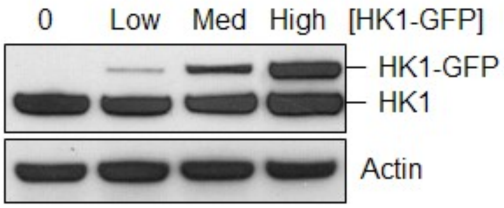

Figure S3. ***Expression of HK1-GFP in NSC34 cells.***

Western blot analysis of total lysate of NSC34 cells transfected with increasing concentration of pEGFPN1-HK1. As the plasmid in the transfection experiment is raised from low to medium to high concentrations, a protein band corresponding to the HK1-GFP appears. The level of endogenous HK1 (lower band recognized by the Ab) was not affected.

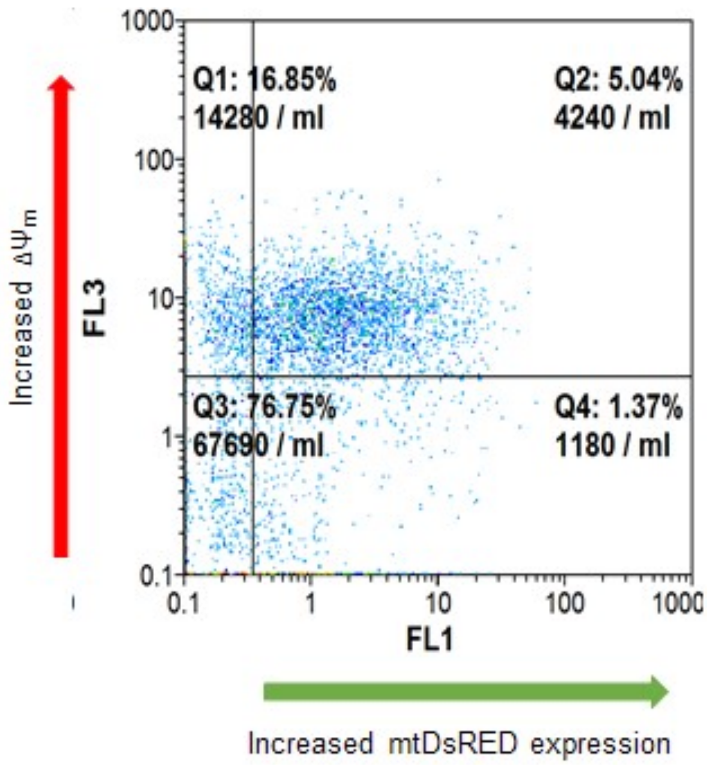

Figure S4. ***ΔΨm analysis in presence of NHK1 peptide*.**

Representative flow cytometry dot plot of NSC34-SOD1G93A cells transfected with NHK1 peptide and stained with TMRM. Q2 and Q4 indicate cells expressing NHK1 peptide, while Q1 and Q3 are not transfected cells (control) form the same population. The transfected cells are mainly placed in the region Q2 corresponding to the highest ΔΨm values, while non transfected cells are equally distributed in Q1 and Q3.

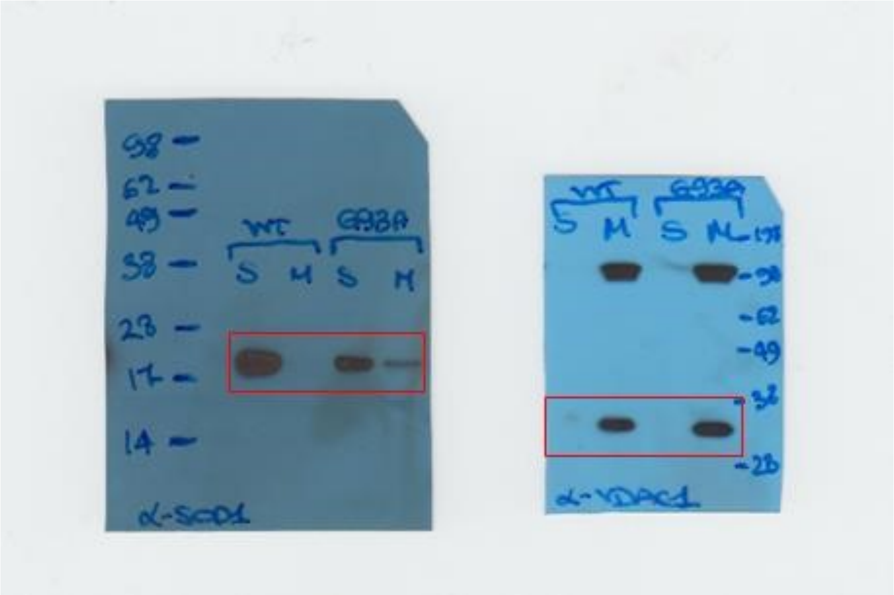

Figure S5. ***Full scans of original western blots showed in Fig. 1A***

In red, the cropped area corresponding to that showed in the main figure. Proteins fractions were loaded in duplicate onto 4-12% NuPage polyacrylamide gel and electro-transferred onto nitrocellulose. Proteins were revealed by using SOD1 antibody (left) or VDAC1 antibody (right).

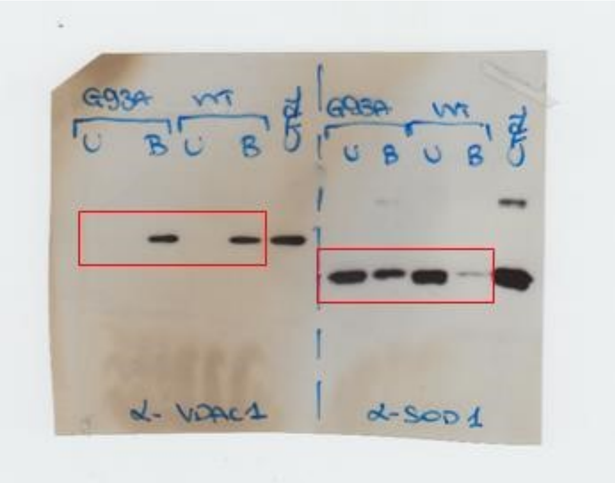

Figure S6. ***Full scans of original western blots showed in Fig. 1B***

In red, the cropped area corresponding to that showed in the main figure. Proteins fractions were loaded in duplicate onto 4-12% NuPage polyacrylamide gel and electro-transferred onto nitrocellulose. Proteins were revealed by using VDAC1 antibody (left) or SOD1 antibody (right). CTRL referred to the purified proteins VDAC1 or SOD1 WT loaded as a control.

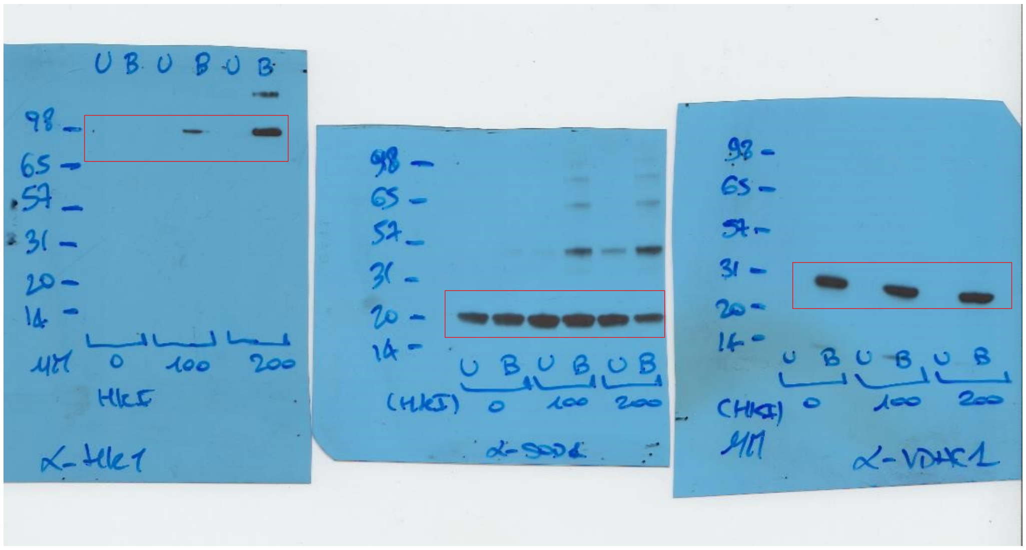

Figure S7. ***Full scans of original western blots showed in Fig. 3A***

In red, the cropped area corresponding to that showed in the main figure. Proteins fractions were loaded in duplicate onto 4-12% NuPage polyacrylamide gel and electro-transferred onto nitrocellulose. Proteins were revealed by using HK1 antibody (left), SOD1 antibody (middle) or VDAC1 antibody (right).

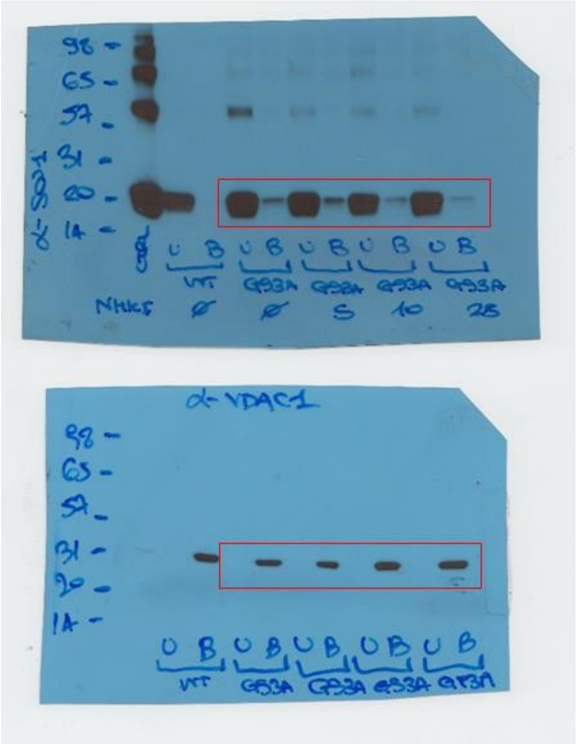

Figure S8. ***Full scans of original western blots showed in Fig. 5A***

In red, the cropped area corresponding to that showed in the main figure. Proteins fractions were loaded in duplicate onto 4-12% NuPage polyacrylamide gel and electro-transferred onto nitrocellulose. Proteins were revealed by using SOD1 antibody (upper) or VDAC1 antibody (lower). CTRL referred to SOD1 WT loaded as a control.

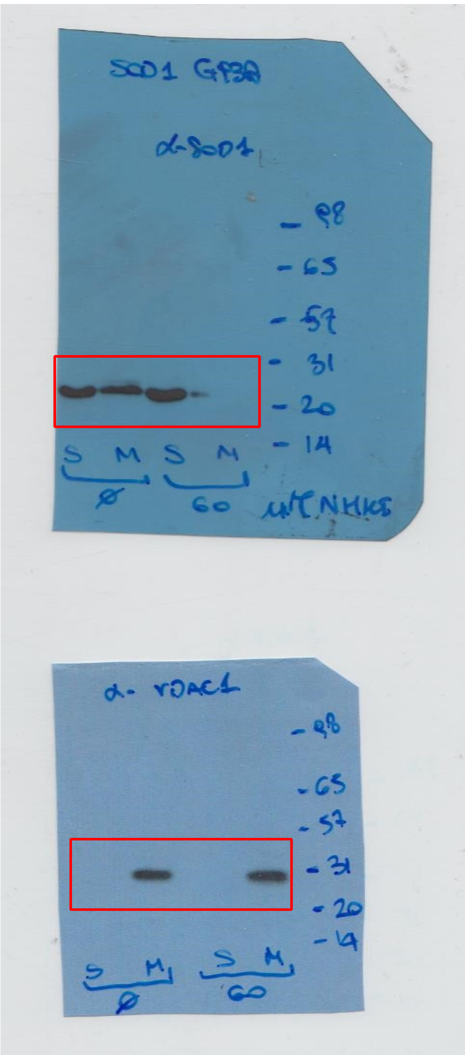

Figure S9. ***Full scans of original western blots showed in Fig. 5B***

In red, the cropped area corresponding to that showed in the main figure. Proteins fractions were loaded in duplicate onto 4-12% NuPage polyacrylamide gel and electro-transferred onto nitrocellulose. Proteins were revealed by using SOD1 antibody (upper) or VDAC1 antibody (lower).

Supplementary References

1. Checchetto, V., Reina, S., Magrì, A., Szabo, I. & De Pinto, V. Recombinant human Voltage Dependent Anion selective Channel isoform 3 (hVDAC3) forms pores with a very small conductance. *Cell Physiol. Biochem.* **34**, 842-853 (2014).

2. Stevens, J. C. *et al.* Modification of Superoxide Dismutase 1 (SOD1) properties by a GFP tag. Implications for research into Amyotrophic Lateral Sclerosis (ALS). *PLoS One* **5**, e9541 (2010).

3. Beauchamp, C. & Fridovich, I. Superoxide dismutase: improved assays and an assay applicable to acrylamide gels. *Anal. Biochem.* **44**, 276-287 (1971).

4. Ferri, A. *et al.* Familial ALS-superoxide dismutases associate with mitochondria and shift their redox potentials. *Proc. Natl. Acad. Sci. U.S.A.* **103**, 13860-13865 (2006).

5. Tomasello, M. F., Guarino, F., Reina, S., Messina, A. & De Pinto, V. The voltage-dependent anion selective channel 1 (VDAC1) topography in the mitochondrial outer membrane as detected in intact cell. *PLoS One* **8**, e81522 (2013).
